# Bufalin Suppresses Colorectal Cancer Liver Metastasis by Inhibiting De Novo Fatty Acid Synthesis via the PI3K/AKT-Mediated SREBP1/FASN Pathway

**DOI:** 10.3390/molecules30173634

**Published:** 2025-09-05

**Authors:** Wenwen Pang, Xiang Li, Suying Yan, Junshi Zhang, Ping Wu, Haiyang Yu, Bowei Zhang, Chunze Zhang

**Affiliations:** 1 Department of Clinical Laboratory, Tianjin Union Medical Center, Nankai University, Tianjin 300071, China; wwpangscu@163.com (W.P.);; 2 Tianjin Integrative Traditional Chinese and Western Medicine Oncology Institute, Tianjin 300121, China; 3 School of Medicine, Nankai University, Tianjin 300071, China; 4 School of Integrative Medicine, Tianjin University of Traditional Chinese Medicine, Tianjin 301617, China; 5 Department of Hematology, Oncology Center, Tianjin Union Medical Center, Nankai University, Tianjin 300071, China; 6 State Key Laboratory of Component-Based Chinese Medicine, Tianjin University of Traditional Chinese Medicine, Tianjin 301617, China; 7 Department of Colorectal Surgery, Tianjin Union Medical Center, Nankai University, Tianjin 300071, China; 8 The Institute of Translational Medicine, Tianjin Union Medical Center, Nankai University, Tianjin 300071, China; 9 Tianjin Institute of Coloproctology, Tianjin 300121, China

**Keywords:** bufalin, colorectal cancer, liver metastasis, de novo fatty acid synthesis, PI3K/AKT-mediated SREBP1/FASN pathway

## Abstract

Background: Colorectal cancer (CRC) is the third most common cancer worldwide, with liver metastasis being the leading cause of mortality. De novo fatty acid synthesis plays a critical role in CRC progression and metastasis. Bufalin, a cardiotonic steroid isolated from toad skin, has demonstrated anticancer activity in multiple preclinical models. However, the mechanisms underlying its suppression of CRC metastasis and modulation of fatty acid synthesis remain to be elucidated. Methods: The effects of bufalin on CRC cell proliferation, migration, and apoptosis were assessed via colony formation, wound healing, and flow cytometry assays. Transcriptome analysis identified bufalin-affected pathways, with changes in gene and protein expression. FASN protein levels were quantified using ELISA. Results: Bufalin inhibited proliferation and migration of CRC cells and induced the apoptosis of LoVo and HCT8 cells. Transcriptome analysis highlighted lipid metabolism pathways as potential mediators of bufalin’s anti-metastatic activity. Notably, bufalin reduced the expression of fatty acid synthase (FASN) and suppressed CRC metastasis. In vivo experiments demonstrated that bufalin attenuated CRC progression and liver metastasis by inhibiting de novo fatty acid synthesis through the PI3K/AKT-mediated SREBP1/FASN pathway. Conclusions: Bufalin inhibits de novo fatty acid synthesis via the PI3K/AKT-mediated SREBP1/FASN pathway, suppressing CRC progression and liver metastasis.

## 1. Introduction

Colorectal cancer (CRC) remains a leading cause of cancer-related mortality worldwide. In recent years, the incidence rate of CRC has notably increased among individuals under 50, reflecting a concerning shift in the burden of disease to younger populations [1]. Approximately 20% have metastatic disease at diagnosis and another 25% have metastasis during treatment [2]. Survival rates for metastatic CRC are extremely low, with only 30–35% of patients surviving more than three years and less than 20% reaching five-year survival [2,3]. Notably, liver metastasis is the most common metastasis site of CRC metastasis [4]. These statistics underscore an urgent need for novel therapies, especially strategies that target liver metastasis.

Recent studies have shown that fatty acid synthesis plays a key role in the progression of malignant cancers [5]. Uncontrolled de novo fatty acid synthesis supplies tumor cells with structural lipids and lipid-derived signals, thereby conferring growth and survival advantages [6]. This induction of lipogenesis is primarily regulated by sterol regulatory element-binding protein 1 (SREBP1), an endoplasmic reticulum membrane-bound protein that functions as a transcription factor for key enzymes, including fatty acid synthase (FASN) [7]. FASN, a critical enzyme in de novo fatty acid synthesis, is significantly elevated in patients with metastatic CRC [8,9]. In CRC, FASN is often overexpressed in metastatic lesions and correlates with disease aggressiveness [8,9]. High expression of FASN in CRC cells can enhance oxidative respiration and confer survival benefits, contributing to therapy resistance and metastatic potential [10]. Therapeutic agents that can block the SREBP1/FASN lipogenic pathway may effectively “starve” cancer cells of crucial lipid resources and prevent metastatic progression. Consequently, inhibition of fatty acid synthesis is considered to be a promising strategy for the treatment of CRC progression and metastasis.

Bufalin (BF), a natural bioactive small molecule derived from toad venom, is a widely used traditional Chinese medicine in cancer treatment, including CRC [11]. Similarly to other cardiotonic steroids such as digoxin and ouabain, bufalin primarily targets the Na/K-ATPase pump and regulates signal transduction pathways [12]. Bufalin has demonstrated anticancer activity against various malignancies, including colorectal, liver, lung, and ovarian cancers [11,12]. Previous studies have shown that bufalin regulates the tumor immune microenvironment by reversing cancer-associated fibroblast-induced CRC invasion and metastasis through the STAT3 pathway [13]. Additionally, bufalin inhibits angiogenesis by targeting the SRC-3/HIF-1α axis, thereby suppressing VEGF-mediated vascular formation and alleviating tumor hypoxia in CRC [14]. It is worth noting that dysregulation of FASN is a hallmark of CRC [10,15,16,17], as tumor growth and survival are largely dependent on de novo lipogenesis [15,16]. Therefore, bufalin is a promising therapeutic agent for CRC, but its mechanism of regulating fatty acid synthesis to inhibit colorectal cancer remains unknown [18]. We therefore hypothesized that bufalin suppresses CRC metastasis by downregulating the PI3K/AKT–SREBP1/FASN axis and, consequently, de novo fatty acid synthesis.

In this study, we examined bufalin’s effects on CRC cell survival, proliferation, and migration using LoVo and HCT8 cell lines. Transcriptomic analysis was subsequently performed to identify key pathways associated with bufalin’s anticancer activity. Furthermore, the CRC liver metastasis model and subcutaneous CRC xenograft mouse model were used to verify the in vivo effects of bufalin on CRC metastasis and to clarify its regulation of de novo fatty acid synthesis. Our results further characterize bufalin’s anti-metastatic activity in CRC, although additional mechanistic validation is required to confirm its therapeutic potential.

## 2. Results

### 2.1. Bufalin Inhibits the Proliferation and Migration of CRC Cells

**Figure 1:**
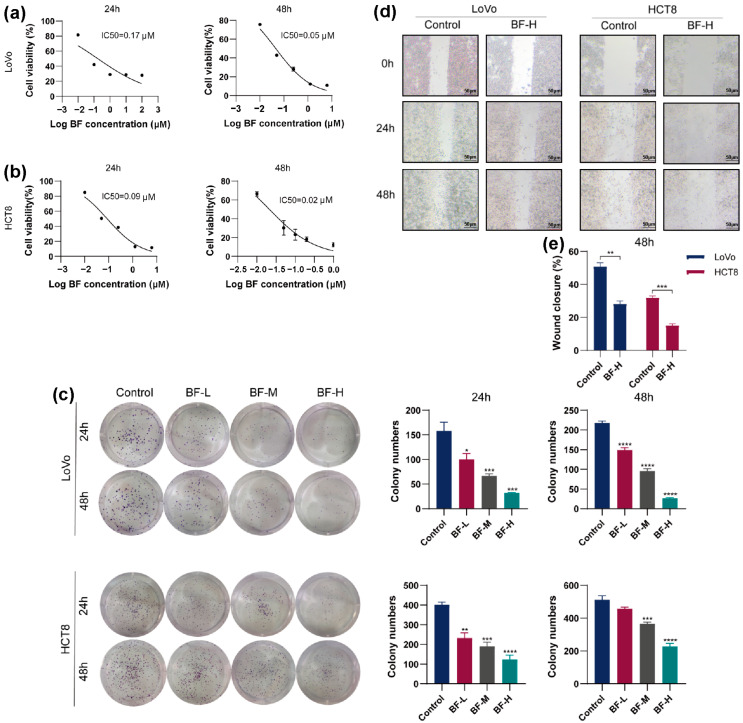
Bufalin inhibited the viability, proliferation, and migration of CRC cells. (**a**) Inhibitory effect of bufalin on LoVo cell viability. (**b**) Inhibitory effect of bufalin on HCT8 cell viability. (**c**) Inhibitory effect of bufalin on the proliferation of CRC cells. (**d**) Inhibitory effect of bufalin on the migration of CRC cells. (**e**) The wound healing rate. The wound closure % was calculated as follows: scratch area of the control group—scratch area of the BF-treated group)/scratch area of the control group × 100%. * *p* < 0.05; ** *p* < 0.01; *** *p* < 0.001; **** *p* < 0.0001.

The effect of bufalin on CRC cell survival was evaluated using a CCK-8 assay. The IC_50_ values of bufalin for LoVo cells were 0.17 μM and 0.05 μM at 24 and 48 h (Figure 1a), respectively, and those of HCT8 cells were 0.09 μM and 0.02 μM (Figure 1b), which is consistent with the results of previous studies [19]. According to the results, the cells in the BF-L, BF-M, and BF-H groups were treated with bufalin at 0.02 μM, 0.05 μM, and 0.1 μM, respectively. To investigate the effect of bufalin on CRC cell proliferation, a colony formation assay was carried out. After treatment with bufalin for 24 or 48 h, colony numbers in LoVo and HCT8 cells were significantly reduced (Figure 1c), showing that bufalin inhibited the proliferation of CRC cells in a dose-dependent manner.

To assess the effect of bufalin on the migration ability of CRC cells, we made scratches on confluent monolayers of LoVo and HCT8 cells, followed by bufalin treatment. Images of the scratch areas were taken at 0, 24, and 48 h, and the wound areas and healing rates were calculated (Figure 1d). By 48 h, the wound healing rate in the BF-H group was significantly decreased in LoVo and HCT8 cells (Figure 1e). Among these groups, the BF-H treatment produced a significantly lower wound healing rate than BF-L, confirming stronger anti-migration activity. Specifically, the wound closure rate of LoVo cells dropped to 27.3% in the BF-H group compared to 51.8% in the control group, suggesting that bufalin significantly inhibited the migration of CRC cells.

### 2.2. Bufalin Induces DNA Damage and Apoptosis

**Figure 2:**
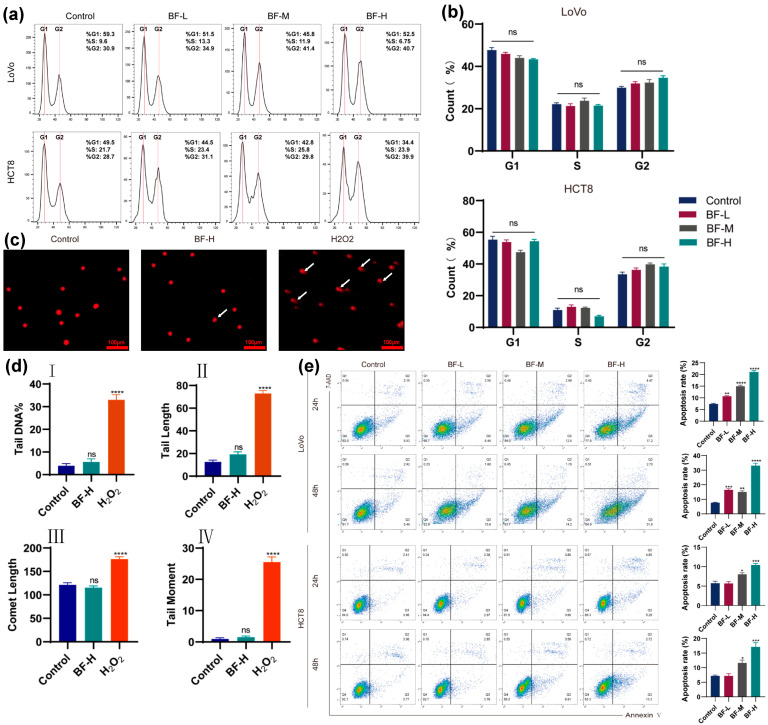
Effects of bufalin on cell cycle, DNA damage, and apoptosis in CRC cells. (**a**) Cell cycle distribution according to flow cytometry. (**b**) Statistical analysis of cell cycle phases. (**c**) Comet assay fluorescence images (scale bar: 100 μm). (**d**) Quantification of DNA damage. (**I**) Percentage of tail DNA, (**II**) tail length (μm), (**III**) comet length (μm), and (**IV**) tail moment for each group. (**e**) Apoptosis detection by flow cytometry. *ns* indicates no statistically significant difference. * *p* < 0.05; ** *p* < 0.01; *** *p* < 0.001; **** *p* < 0.0001.

Abnormal cell cycle progression is a fundamental mechanism in tumorigenesis. Based on transcriptomic sequencing results, we investigated the changes in cell cycle distribution in CRC cells before and after bufalin treatment. LoVo and HCT8 cells were treated with various concentrations of bufalin, and the cell cycle phases were evaluated by flow cytometry. As shown in Figure 2a,b, in LoVo cells, the proportion of cells in the G1 phase slightly decreased in the BF-treated group, while the proportion in the G2 phase was slightly higher than that in the control group. However, these differences were not statistically significant. Similarly, no significant changes in cell cycle distribution were observed in HCT8 cells following bufalin treatment. These findings suggested that bufalin has little impact on the cell cycle progression of CRC cells, implying that the anticancer effect of bufalin is probably mediated not by the regulation of the cell cycle but through other pathways.

To assess DNA damage in CRC cells after bufalin treatment, we performed the comet assay (Figure 2c). In this assay, DNA damage results in smaller DNA fragments that migrate faster during electrophoresis, forming a comet-like tail visible under a microscope [20]. As shown in Figure 2d, comet tail length, the percentage of tail DNA, comet length, and tail moment were significantly increased in the H_2_O_2_ group (positive control) compared to those in the control group. Nevertheless, no obvious tailing phenomenon was observed in the control or BF-H group, and statistical analysis revealed no differences in comet tail length, the percentage of tail DNA, comet length, or tail moment between the control and BF-H groups, suggesting that bufalin did not cause significant DNA damage in CRC cells.

To evaluate the effect of bufalin on cell apoptosis, we used flow cytometry to measure the proportion of apoptotic cells following treatment with different concentrations of bufalin. As shown in Figure 2e, bufalin treatment significantly increased the proportion of apoptotic cells. After exposure to bufalin for 48 h, the apoptotic rates of LoVo cells remarkably increased from 16.41% to 33.14% when the concentration increased from 0.02 μM (BF-L group) to 0.1 μM bufalin (BF-H group). In contrast, the apoptosis rate of the control group was only 7.69%. In addition, after 48 h of bufalin treatment, the apoptotic rates in BF-M group and BF-H group were significantly increased to 11.85% and 17.28%, respectively. In the wound healing assay, BF-H reduced scratch closure to a greater extent than BF-L. These findings indicated that bufalin could induce apoptosis of CRC cells, suggesting bufalin as a candidate for the treatment of CRC.

### 2.3. Transcriptomic Analysis Reveals the Mechanism of Bufalin Against CRC Metastasis

**Figure 3:**
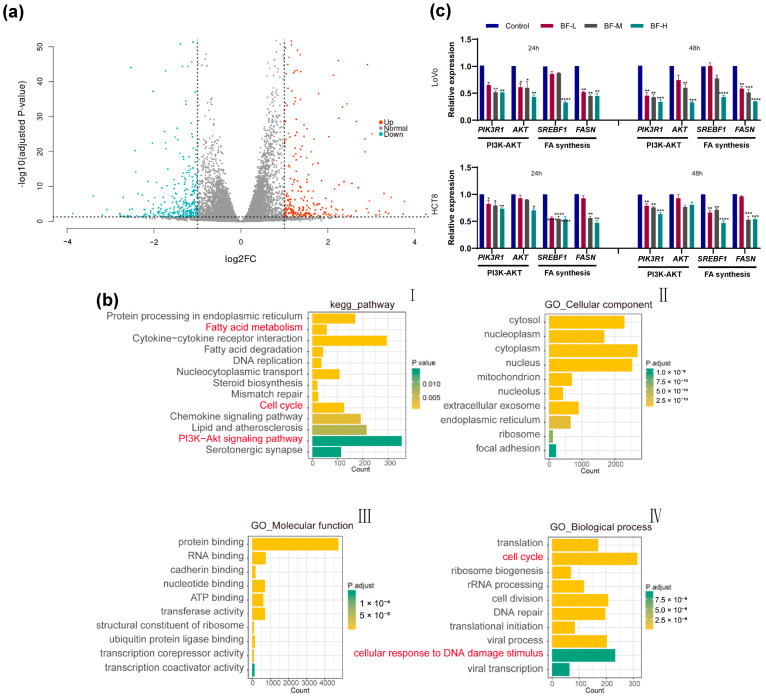
Transcriptomic analysis of bufalin’s effects on CRC cells. (**a**) A volcano plot of DEGs. The volcano plot displays DEGs between the BF-treated group and the control group. FC (fold change) represents the ratio of signal values between the bufalin and CT groups, while Log2FC indicates the logarithm base 2 of this fold change. “Up” denotes upregulated genes, and “Down” indicates downregulated genes. (**b**) Pathway and functional analysis of DEGs. (**I**) Pathway enrichment analysis of DEGs. (**II**) Cellular component analysis of DEGs. (**III**) Molecular function analysis of DEGs. (**IV**) Biological process analysis of DEGs. (**c**) The effects of bufalin on de novo fatty acid synthesis and PI3K-AKT pathway genes in CRC cells. *ns* indicates no statistically significant difference. * *p* < 0.05; ** *p* < 0.01; *** *p* < 0.001; **** *p* < 0.0001.

Given the multi-target and multi-pharmacological properties of traditional Chinese medicine compounds, we employed transcriptome sequencing to examine the transcriptomic profile of HCT8 cells before and after bufalin treatment using high-throughput sequencing. By comparing gene expression profiles between BF-treated and control groups, we identified 2014 differentially expressed genes (DEGs), of which 1252 genes were upregulated and 762 genes were downregulated (Figure 3a).

Further analysis of these DEGs revealed significant effects of bufalin on various biological processes and signaling pathways. In addition, KEGG pathway enrichment analysis revealed substantial changes in pathways related to fatty acid synthesis, DNA replication, cell cycle regulation, chemokine signaling, and the PI3K-AKT signaling pathway in BF-treated cells (Figure 3(bI)). To further elucidate the functional roles of these DEGs, GO enrichment analysis categorized them into cellular components, molecular functions, and biological processes. BF-treated cells displayed marked enrichment in processes such as translation, cell cycle control, and DNA repair, particularly in response to DNA damage stimuli (Figure 3(bII–bIV)).

To validate the transcriptomic findings, we used RT-PCR to measure the expression of *FASN*, a key enzyme in fatty acid synthesis. As shown in Figure 3c, *FASN* expression was significantly downregulated in both LoVo and HCT8 cells following bufalin treatment, except for HCT8 cells in the BF-L group. These results suggested that bufalin effectively inhibited de novo fatty acid synthesis by targeting critical regulators such as *FASN*. Additionally, we observed significant downregulation of *SREBF1*, a major transcriptional regulator of *FASN*, in both cell lines, especially with a more significant effect in the BF-H group.

Furthermore, the PI3K-AKT signaling pathway, a known link between growth factor signaling and FASN expression [21], was markedly suppressed by bufalin. RT-PCR analysis confirmed that the expressions of *PIK3R1* and *AKT* genes were downregulated in both LoVo and HCT8 cells, with a more significant effect observed in LoVo cells (Figure 3c). These findings suggested that bufalin might inhibit the progression of cancer cells by inhibiting de novo fatty acid synthesis through the PI3K-AKT pathway.

### 2.4. Bufalin Inhibits De Novo Fatty Acid Synthesis Through the PI3K-AKT Pathway

**Figure 4:**
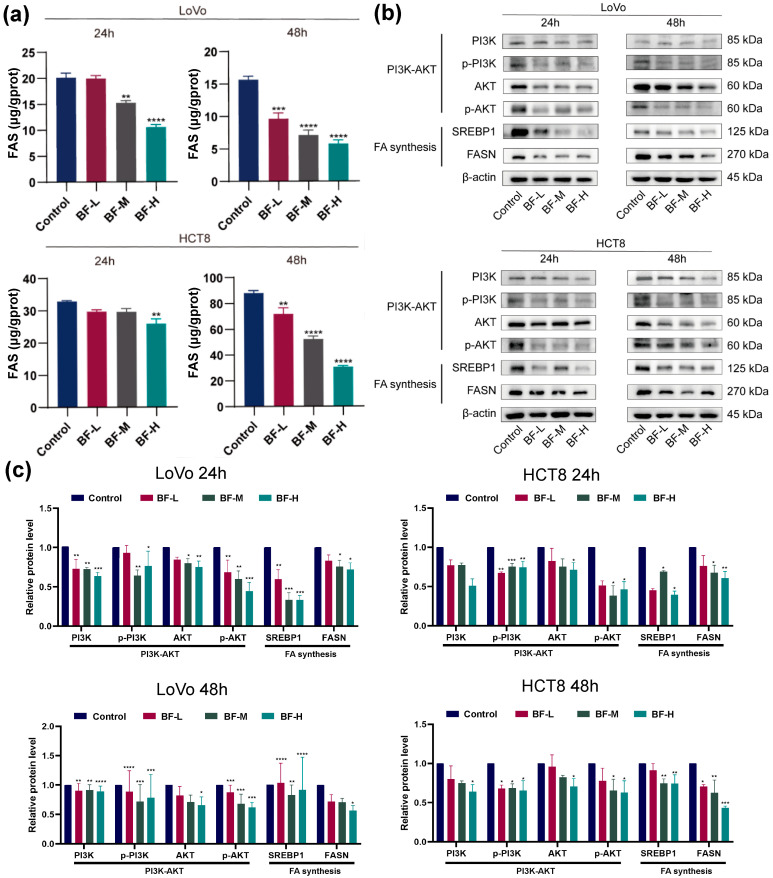
Effects of bufalin on de novo fatty acid synthesis pathway in CRC cells. (**a**) FASN levels in CRC cells after bufalin treatment. (**b**) Western blot analysis of CRC cells after bufalin treatment. (**c**) Protein expression of PI3K/AKT-mediated SREBP1/FASN signaling pathways in CRC cells. * *p* < 0.05; ** *p* < 0.01; *** *p* < 0.001; **** *p* < 0.0001.

Based on transcriptomic analysis and the above results, we further investigated the effects of bufalin on fatty acid synthesis, with special attention to FASN, a key enzyme in de novo fatty acid synthesis. Intracellular fatty acid synthase levels were measured using ELISA, which revealed a significant reduction in FASN levels in both LoVo and HCT8 cells following bufalin treatment (Figure 4a). The FASN levels of LoVo cells decreased significantly from 9.65 μg g^−1^ protein to 5.78 μg g^−1^ protein when bufalin concentrations increased, compared to 15.67 μg g^−1^ protein in the control group after 48 h of treatment. These findings indicate that bufalin effectively inhibits CRC metastasis by reducing FASN levels and inhibiting de novo fatty acid synthesis.

Notably, bufalin modulates the PI3K-AKT signaling pathway, which plays an indispensable role in connecting growth factor signaling with FASN expression. Western blot analysis showed that in BF-treated cells, both the total and phosphorylated levels of PI3K and AKT were significantly reduced (Figure 4b). The decrease in phosphorylation levels indicated that bufalin inhibited the activation of the PI3K-AKT pathway, thereby reducing FASN expression and lipid synthesis. SREBP1 protein levels also decreased following bufalin treatment, further confirming the role of this pathway in the inhibition of fatty acid synthesis in CRC cells by bufalin (Figure 4c). Collectively, our data indicate that bufalin suppresses de novo lipogenesis via the PI3K/AKT-SREBP1/FASN axis, which in turn limits CRC cell proliferation and motility.

### 2.5. Bufalin Inhibits CRC Progression in Mouse Models

**Figure 5:**
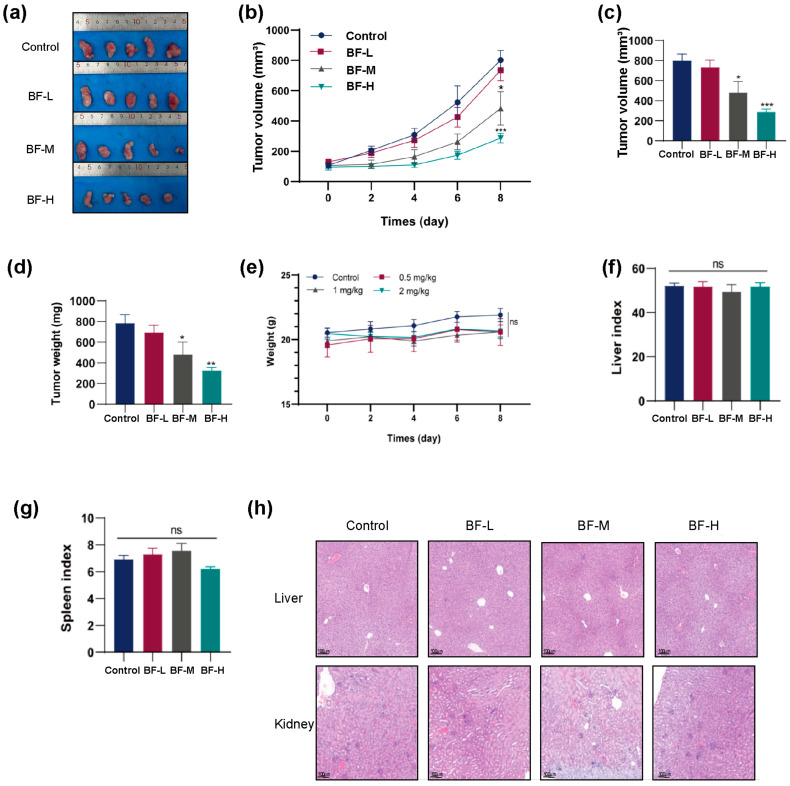
Bufalin inhibited the CRC progression in mouse models. (**a**) Images of subcutaneous tumors in each group at the end of the experiment. (**b**) Tumor growth curves showing changes in tumor volume over time. (**c**) Tumor volumes at the end of the experiment. (**d**) Tumor weights at the end of the experiment. (**e**) Growth curves of body weight. (**f**) Liver index. (**g**) Spleen index. (**h**) H&E-stained liver and kidney tissue sections imaged at 100× magnification (scale bar: 200 μm). * *p* < 0.05; ** *p* < 0.01; *** *p* < 0.001.

Mice in the subcutaneous xenograft model received daily intraperitoneal injections of bufalin and were monitored daily for tumor growth (Figure 5a), with body weight and tumor volume recorded every other day. On day 8 of treatment, the maximum tumor volume in the control group approached 1000 mm^3^, at which point drug administration was stopped, and the experiment was concluded (Figure 5b). As shown in Figure 5c,d, both tumor volume and weight were significantly lower in the BF-L, BF-M, and BF-H groups than those in the control group. Specifically, we found that the tumor volume in the BF-H group was significantly reduced by about four times compared to that in the control group (Figure 5c), suggesting that bufalin significantly inhibited the development and progression of CRC in vivo.

Safety evaluation was performed, including body weight, the liver index, the spleen index, and H&E staining of major organs. As shown in Figure 5e, there was no difference in body weight between the four groups. We measured the weight of the liver and spleen and calculated the organ indices. The results showed that bufalin treatment did not affect the liver index or spleen index (Figure 5f,g). No evident histopathological abnormalities were observed in either the liver or the kidney (Figure 5h). Therefore, bufalin could be considered as a safe and innovative treatment option for CRC and CRC metastasis.

### 2.6. Bufalin Inhibits CRC Liver Metastasis in Mouse Models

**Figure 6:**
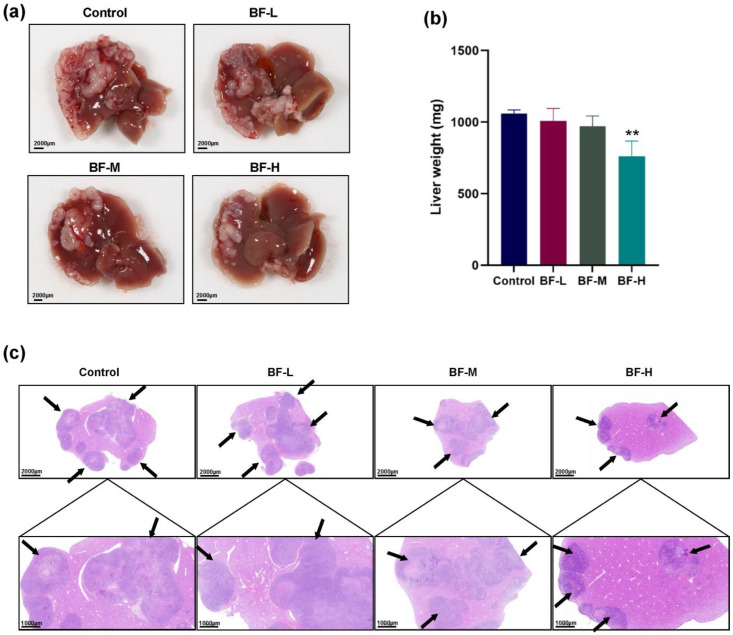
Bufalin alleviated the malignant degree of CRC liver metastasis. (**a**) Representative images of liver tissues from mice with CRC liver metastasis and the corresponding quantitative results. (**b**) The liver weight of mice. (**c**) H&E-stained liver tissue sections imaged at 10 × and 20× magnification (scale bar: 2000 μm or 1000 μm). ** *p* < 0.01.

To verify the inhibitory effect of bufalin on CRC metastasis, which was demonstrated in cell migration experiments in vitro, we validated this in vivo using a mouse model of CRC liver metastasis. The spleen-to-liver metastasis model of CRC was established, and bufalin was administered as treatment as described. Then, we observed the presence of metastatic lesions in the liver and examined characteristics such as the size, number, location, morphology of the lesions and their relationships with the surrounding tissues. To assess the degree of CRC liver metastasis, the weight of the liver was used to evaluate the tumor size. The results showed that the degree of CRC liver metastasis was significantly alleviated in the BF-H group. Most notably, it can be seen that bufalin significantly reduced the number of CRC metastatic nodules (Figure 6a), indicating effective suppression of liver metastasis in CRC by bufalin. As shown in Figure 6b, the liver weight was significantly reduced to 760.2 mg in the BF-H group compared to 1060.8 mg in the model group.

H&E staining showed irregular hepatocytes with enlarged, hyperchromatic nuclei in control mice, whereas bufalin-treated livers displayed near-normal morphology. It is worth noting that after bufalin treatment, tumor cell density and irregular cells were decreased (Figure 6c), suggesting that bufalin significantly alleviated the malignant degree of CRC liver metastasis.

### 2.7. Bufalin Inhibits De Novo Fatty Acid Synthesis Through the PI3K/AKT-Mediated SREBP1/FASN Signaling Pathway

**Figure 7:**
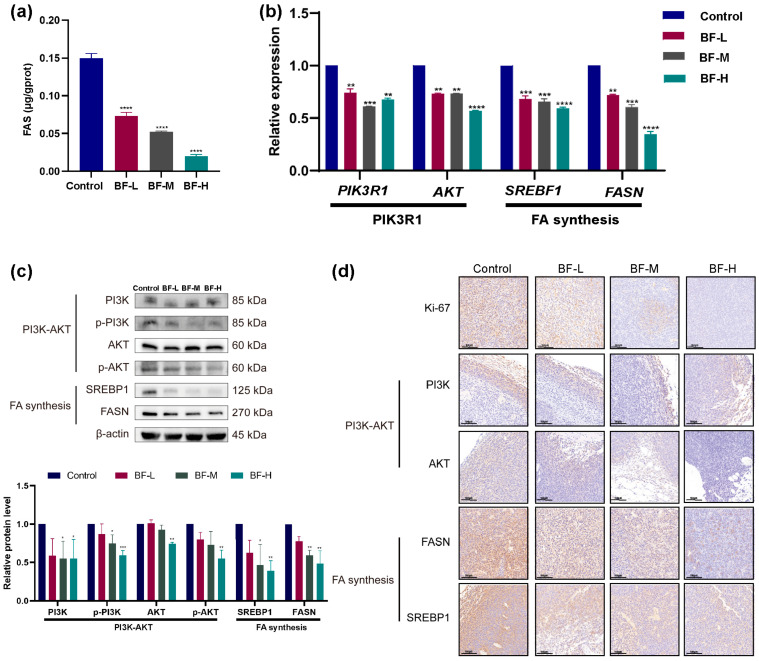
Effects of bufalin on PI3K/AKT-mediated SREBP1/FASN signaling pathways in mice. (**a**) Effects of bufalin on FASN levels in tumor tissue. (**b**) Effects of bufalin on mRNA levels of *PIK3R1*, *AKT*, *SREBF1*, and *FASN* genes in tumor tissue. (**c**) Effects of bufalin on protein levels of PI3K, AKT, SREBP1, and FASN in tumor tissue. (**d**) IHC images of Ki-67, PI3K, AKT, SREBP1, and FASN. The IHC sections are imaged at 200× magnification (scale bar: 100 μm). * *p* < 0.05; ** *p* < 0.01; *** *p* < 0.001; **** *p* < 0.0001.

To investigate the effects of bufalin on fatty acid synthesis in vivo, we used an ELISA kit to measure FASN content in tumor tissues. As shown in Figure 7a, the FASN levels in the BF-L, BF-M, and BF-H groups were decreased to 0.073 μg g^−1^ protein, 0.052 μg g^−1^ protein, and 0.020 μg g^−1^ protein, respectively. Bufalin treatment significantly reduced FASN levels in a dose-dependent manner compared to the control, indicating that bufalin inhibited de novo fatty acid synthesis in the CRC mouse model.

We further quantified *PIK3R1*, *AKT*, *SREBF1*, and *FASN* mRNA levels by RT-qPCR. As shown in Figure 7b, these genes were significantly downregulated in the BF-treated groups. Western blot analysis further assessed the protein levels of FASN, SREBP1, and PI3K-AKT pathway components in tumor tissues (Figure 7c). The total PI3K protein levels decreased in the BF-M and BF-H groups, accompanied by a marked reduction in phosphorylation levels. In the BF-H group, both AKT protein and phosphorylation levels were significantly decreased. Similarly, SREBP1 and FASN protein levels were significantly downregulated in the BF-M and BF-H groups.

To further verify the effect of bufalin on the PI3K/AKT-mediated SREBP1/FASN signaling pathways, immunohistochemistry (IHC) staining was performed on tumor tissues (Figure 7d). IHC analysis showed that the expression of Ki-67, a marker of tumor proliferation, was significantly reduced in a dose-dependent manner. Additionally, IHC staining confirmed that FASN and SREBP1 protein levels in BF-treated groups were significantly reduced compared with the control in a dose-dependent manner. The PI3K and AKT expression levels were also decreased after bufalin treatment.

These results suggest that bufalin effectively inhibits CRC progression and metastasis by modulating lipid metabolism via the PI3K/AKT signaling pathway and its downstream target, SREBP1. While further studies are warranted to explore additional regulatory mechanisms, our results provide strong evidence that targeting de novo lipogenesis through PI3K/AKT-SREBP1/FASN modulation represents a potential therapeutic approach for CRC.

## 3. Discussion

Metastatic CRC remains a significant clinical challenge, with liver metastases contributing to poor prognosis. Effective strategies to suppress metastasis are crucial to improve survival in CRC patients [22]. In recent years, traditional Chinese medicine has played an important role as an adjunct and complementary therapy in cancer treatment, demonstrating potential in preventing and managing CRC metastasis [23]. Bufalin, a cardiotonic steroid derived from toad venom, has demonstrated broad anti-tumor properties [11,23], but its effects on CRC metastasis, particularly via metabolic pathways, remain underexplored. In our study, we confirmed that bufalin inhibits CRC progression and liver metastasis. Consistently with prior reports on other CRC cell lines [24,25,26], bufalin significantly inhibited the viability and clonogenic growth of LoVo and HCT8 CRC cells. Given that tumor cell migration is a critical phenotype of cancer progression and a rate-limiting step in metastasis, we performed scratch assays to evaluate the anti-metastatic activity of bufalin. The in vivo results showed that bufalin treatment not only inhibited the growth of subcutaneous tumor but also suppressed CRC liver metastasis. Compared with the untreated control group, the mice treated with bufalin produced significantly fewer and smaller metastatic liver nodules, indicating that metastatic colonization was significantly inhibited. In addition, bufalin’s anti-tumor doses in mice did not cause obvious toxicities. Overall, the results of the in vitro and in vivo studies found bufalin to be an effective Chinese medicine for the treatment of CRC progression and metastasis, supporting its promise as a therapeutic candidate against metastatic CRC.

Previous studies have linked bufalin to various anticancer mechanisms such as apoptosis induction, autophagy, anti-angiogenesis and modulation of the tumor microenvironment. In our CRC models, however, bufalin did not measurably alter cell cycle distribution, suggesting that its anti-metastatic activity is driven mainly by metabolic reprogramming and apoptosis rather than cell cycle blockade [11,18,27,28,29]. To elucidate the mechanisms underlying bufalin’s anti-metastatic effects in CRC, we employed transcriptome sequencing to identify DEGs in CRC cells before and after bufalin treatment. These findings imply that bufalin inhibits CRC progression primarily by suppressing fatty acid biosynthesis and attenuating PI3K–AKT signaling; effects on cell cycle distribution appear minimal. These transcriptomic changes did not correspond to functional alterations in cell cycle phases, implying that post-transcriptional regulation or pathway crosstalk may buffer the phenotypic impact. While bufalin has been reported to induce cell cycle arrest in certain cancers [30,31,32], our results did not show that bufalin has an effect on cell cycle progression in CRC. In addition, we observed that bufalin induced apoptosis, in line with previous reports that bufalin, can activate intrinsic apoptotic pathways [24]. Notably, the transcriptome sequencing results provide important clues that, in addition to its known role, bufalin may affect tumor metabolic pathways, particularly fatty acid synthesis, which has not been previously associated with its action in CRC. Based on this, the mechanism of bufalin against CRC liver metastasis was further investigated.

Enhanced de novo fatty acid synthesis, characterized by the upregulation of several lipogenic enzymes, serves as a hallmark of numerous tumors [33,34]. FASN, the enzyme responsible for catalyzing the final steps of de novo fatty acid synthesis, is typically overexpressed in cancer cells but maintained at low levels in normal tissues [8]. Inhibition of FASN can induce cancer cell death through mechanisms such as membrane disruption, inhibition of DNA replication, accumulation of toxic malonyl-CoA, and suppression of anti-apoptotic proteins [8,33]. Recent studies have strengthened the link between aberrant lipid metabolism and CRC aggressiveness. Reprogramming of lipid metabolism in cancer-associated fibroblasts was shown to enhance CRC cell migration [5], and overexpression of FASN in CRC cells increases O-GlcNAcylation of proteins, promoting tumor growth and survival [35]. Before our work, bufalin’s influence on tumor lipid metabolism was unknown. Our data demonstrate for the first time that bufalin significantly downregulates FASN in vitro and in vivo CRC models. We also observed an accompanying downregulation of SREBP1, an upstream transcription factor that drives FASN expression. Given the dual role of SREBP1 in both fatty acid and cholesterol synthesis, future work will investigate whether bufalin also affects cholesterol metabolism and its potential impact on CRC metastasis. It is worth noting that the previously reported anti-metastatic mechanisms of bufalin in CRC involved inhibition of the STAT3 pathway, etc. [13]. Our findings reveal an additional, novel mechanism—the suppression of the SREBP1/FASN lipogenesis pathway. Because bufalin suppresses fatty acid biosynthesis, it may reduce the lipid resources required for tumor cell proliferation and metastatic colonization. This mechanistic insight adds a new dimension to bufalin’s anti-CRC activity and provides a rationale for combining bufalin with other treatments that target cancer metabolism.

Additionally, we found that bufalin modulates the SREBP1/FASN pathway via the PI3K/AKT signaling pathways, which is a major regulator of cellular metabolism and tumor progression [19,36]. Bufalin treatment led to reduced PI3K and AKT phosphorylation, alongside decreased SREBP1 and FASN expression, both in vitro and in vivo. This suggests that bufalin impairs CRC progression and liver metastasis via metabolic disruption. Specifically, bufalin inhibits fatty acid synthesis, by targeting the PI3K/AKT-mediated SREBP1/FASN signaling pathways. These findings position bufalin as a metabolic inhibitor with anti-metastatic potential.

## 4. Materials and Methods

### 4.1. Chemicals and Reagents

Bufalin was purchased from Shanghai Taoshu Biotechnology (Shanghai, China). RPMI-1640 medium, fetal bovine serum (FBS), trypsin-EDTA, penicillin-streptomycin, and DMSO were obtained from Gibco (Thermo Fisher Scientific Inc., Waltham, MA, USA). The CCK-8 assay kit was sourced from Absin Bioscience Inc. (Shanghai, China). The cell cycle and Annexin V-FITC/PI Apoptosis Detection Kit were purchased from KeyGen Biotech (Nanjing, China). Enzyme-linked immunosorbent assay (ELISA) kits for FASN detection were acquired from Abcam (Cambridge, UK). The SteadyPure quick RNA extraction kit and the SYBR green premix Pro Taq HS qPCR kit were obtained from Accurate Biology (Guangzhou, China). The RIPA lysis buffer and BCA protein assay kit were acquired from Beyotime (Shanghai, China). The antibodies for FASN, PI3K, AKT, and SREBP1 were purchased from WanleiBio Co., Ltd. (Shenyang, China).

### 4.2. Cell Culture

Human CRC lines LoVo and HCT8 were provided by the Shanghai Institute of Life Sciences, Chinese Academy of Sciences. Cells were cultured in RPMI-1640 medium supplemented with 10% FBS and 1% penicillin–streptomycin and maintained at 37 °C in a humidified incubator with 5% CO_2_.

### 4.3. Cell Viability Assay

The cells were seeded into 96-well plates at a density of 5000 cells per well and treated with different concentrations of bufalin for 24 or 48 h. Following treatment, CCK-8 reagent was added, and the cells were incubated for 1 h. Optical density (OD) was measured at 450 nm with a microplate reader to determine cell viability.

### 4.4. Colony Formation Assay

The cells were seeded into the six-well plate with 700 cells per well and treated with different concentrations of bufalin for 24 h. The fresh medium containing bufalin or not was changed every three days. After treatment for 10 days, the cells were fixed with 4% paraformaldehyde and stained with 0.5% crystal violet. Colonies containing more than 50 cells were counted under microscope.

### 4.5. Wound Healing Assay

The cells were seeded into a six-well plate. When cells grew to 80%, scratches were generated using a 200 μL pipette tip, and fresh medium containing 0.1 μM bufalin was added. Then CRC cells were subsequently washed and treated with bufalin in serum-free medium. The wound closure was monitored and imaged at 0, 24, and 48 h. The migration rate was calculated using ImageJ 1.8.0.

### 4.6. Cell Cycle and Apoptosis Analysis

To analyze cell cycle distribution, the cells were treated with different concentrations of bufalin for 48 h. Then, cell cycle and apoptosis analysis were performed as described before [37]. In cell cycle analysis, propidium iodide (PI) was used to stain the DNA content of cells. The DNA content varies in each stage of the cell cycle, and appropriate gates were set to segregate these phases according to their characteristic DNA content distributions.

In apoptosis analysis, Annexin V-FITC and propidium iodide (PI) staining were utilized. Gates were established to distinguish between viable cells (Annexin V-negative and PI-negative), early apoptotic cells (Annexin V-positive and PI-negative), and late apoptotic cells (Annexin V-positive and PI-positive). The cells treated with bufalin were collected for staining and analysis using the BD FACSCanto II flow cytometer and FlowJo 10.8.1.

### 4.7. Comet Assay

DNA damage was evaluated using the comet assay. The cells were embedded in an agarose gel, lysed, and subjected to electrophoresis under alkaline conditions. The slide was stained with ethidium bromide, and comet tails were observed under a fluorescence microscope. Tail length, the percentage of DNA in the tail, and tail moment were quantified to evaluate DNA damage.

### 4.8. RNA Sequencing

RNA sequencing was conducted to investigate the transcriptome changes induced by bufalin in CRC cells. Total RNA was extracted from HCT8 cells treated with bufalin and untreated control cells using TRIzol reagent. The RNA was subjected to quality control, including concentration and integrity assessment. High-throughput RNA sequencing was performed on the NovaSeq XP System (Illumina, San Diego, CA, USA). Raw data were processed to remove adapters and low-quality reads and then aligned to the reference genome. Differential gene expression analysis identified significantly upregulated and downregulated genes with |log2FC| > 1 and FDR < 0.05. In addition, KEGG and GO databases were used for enrichment analysis and plotting.

### 4.9. ELISA for FASN Detection

To assess the effect of bufalin on fatty acid synthesis, FASN levels in CRC cells and tumor tissues were determined. The FASN levels were quantified using an ELISA kit according to the manufacturer’s protocol, and absorbance was measured at 450 nm.

### 4.10. Quantitative Real-Time PCR

The total RNA was reverse-transcribed into cDNA. Then, RT-PCR was performed using SYBR Green to quantify the expression of *PIK3R1* (a regulatory subunit of PI3Ks), *AKT*, *SREBF1* (sterol regulatory element binding transcription factor 1 Gene), and *FASN* genes. The primer sequences of these genes are shown in the Supplementary Materials. The relative expression levels were normalized to β-actin and analyzed using the 2^−ΔΔCt^ method (ΔCt = Ct_target gene_ − Ct_β-actin_; ΔΔCt = ΔCt_treated group_ − ΔCt_control group_).

### 4.11. Western Blot

Protein samples were extracted from cells and tumor tissues using RIPA lysis buffer supplemented with protease and phosphatase inhibitors. The concentration of proteins was determined using a BCA protein assay kit. Equal amounts of protein were separated by SDS-PAGE and then transferred to PVDF membranes. The membranes were blocked with 5% non-fat milk in TBST at 37 °C for 1 h to prevent non-specific binding. We diluted the corresponding antibodies in the following proportions: PI3K (1:500), AKT (1:1000), p-PI3K (1:500), p-AKT (1:2000), SREBP1 (1:2000), and FASN (1:2000). After blocking, the membranes were incubated with specific primary antibodies at 4 °C overnight. The next day, the membranes were washed three times with TBST and then incubated with secondary antibodies (1:10,000 dilution) at room temperature for 1 h. After washing the membranes thoroughly with TBST, the protein bands were visualized and imaged using an ECL detection system. For band quantification, we analyzed the optical density of bands in the image using ImageJ software.

### 4.12. Animal Model for CRC and CRC Metastasis

All procedures were conducted in accordance with the “Guiding Principles in the Care and Use of Animals” (China) and were approved by the Tianjin Union Medical Center (2023-B30). The BALB/c nude mice used in this study were from Beijing Vital River Laboratory Animal Technology Co., Ltd. (Beijing, China). To establish the CRC model, the LoVo cells (2 × 10^6^ cells/200 μL) were injected subcutaneously into the right flank and were then randomly grouped using the lottery method. Based on previous research, the control group received a daily intraperitoneal injection of 50 μL PBS, while the BF-L, BF-M, and BF-H treatment groups (*n* = 8 per group) received daily intraperitoneal injections of bufalin in PBS at doses of 0.5 mg/kg, 1 mg/kg, and 2 mg/kg, respectively (50 μL per injection) [38]. Drug preparation and administration were performed by investigators blinded to group allocation, while outcome measurements were conducted by an independent, similarly blinded team. Treatment was continued until tumors reached 1000 mm^3^. Specifically, the animals were euthanized after eight days of treatment and the study ended. Tumor dimensions (length and width) were measured, and tumor volume (V, mm^3^) was calculated using the formula V = 0.5 × L × W^2^.

For the CRC metastasis model, LoVo cells (5 × 10^6^ cells/100 μL) were directly injected into the spleen according to a previous study [39]. The treatment was started one week after injection. The control and BF-treated groups were intraperitoneally injected with PBS or 2 mg/kg bufalin for 21 days, respectively. The presence of metastatic lesions in the liver and examined characteristics such as the size, number, location, and morphology of the lesions were observed. To assess the degree of CRC liver metastasis, the livers with CRC liver metastasis were weighed. At the end of the experiment, the mice were anesthetized with tribromoethanol and euthanized via cervical dislocation. Subsequently, tumor tissues, liver tissues, and liver–kidney tissues were collected. The liver index and spleen index were calculated based on organ-to-body-weight ratios.

### 4.13. Hematoxylin–Eosin (H&E)

Tissue samples were fixed in 4% (*w*/*v*) paraformaldehyde at room temperature for 24 h, followed by dehydration in a graded ethanol series and embedding in paraffin. Paraffin-embedded tissues were sectioned into 5 μm slices using a microtome. For H&E staining, sections were deparaffinized in xylene, rehydrated through graded ethanol, and washed with PBS. Hematoxylin was applied for 5 min, followed by eosin staining for 2 min. Then, the HE-stained tissue sections are ready for microscopic examination.

### 4.14. Immunohistochemistry

Fresh tissues were fixed in 10% formalin, dehydrated, embedded in paraffin, and sliced. The sections were first deparaffinized and rehydrated, followed by antigen retrieval and blockage of endogenous peroxidase activity. Tissue sections were then blocked with 5% bovine serum albumin for 30 min. We diluted the corresponding primary antibodies at the following ratios: Ki-67 (1:150), PI3K (1:200), AKT (1:300), SREBP1 (1:200), and FASN (1:200). The primary antibodies were applied to the sections and incubated overnight at 4 °C. After being washed by PBS, the sections were incubated with HRP-conjugated secondary antibodies for 30 min at room temperature. The DAB substrate was used for color development, followed by counterstaining with hematoxylin. After dehydration and mounting, the stained sections were observed and imaged using a Zeiss microscope. We calculated the percentage of positively stained cells in at least three randomly selected fields per section for quantification.

### 4.15. Statistical Analysis

All experiments were repeated three independent times for statistical analysis. Data were expressed as the mean ± standard error of the mean (SEM). One-way analysis of variance (ANOVA) was used for statistical significance assessment, followed by Tukey’s post hoc test for multiple comparisons. Prior to ANOVA, the normality of data was assessed using the Shapiro–Wilk test, and the homogeneity of variance was confirmed with Levene’s test. A *p*-value < 0.05 was considered statistically significant.

## 5. Conclusions

Our results demonstrate that bufalin modulates tumor metabolic pathways, thereby inhibiting CRC growth and metastasis. By downregulating the PI3K/AKT-mediated SREBP1/FASN pathway, bufalin effectively suppresses de novo fatty acid synthesis, which is critical for CRC progression and liver metastasis. The mechanism reported in this study complements the pharmacological profile of bufalin and highlights its potential as a traditional Chinese medicine for the treatment of metastatic CRC.

## Data Availability

The raw data of transcriptome sequencing presented in this study can be found in online repositories. Accession code to cite these SRA data: PRJNA1225464.

## Supplementary Materials

The following supporting information can be downloaded at: https://www.mdpi.com/article/10.3390/molecules30173634/s1.

## Abbreviations

AKT	Protein Kinase B
BF	Bufalin
CCK-8	Cell Counting Kit-8
CRC	Colorectal Cancer
DEGs	Differentially Expressed Genes
DMSO	Dimethyl Sulfoxide
ELISA	Enzyme-Linked Immunosorbent Assay
FASN	Fatty Acid Synthase
GO	Gene Ontology
H&E	Hematoxylin and Eosin
IHC	Immunohistochemistry
KEGG	Kyoto Encyclopedia of Genes and Genomes
PBS	Phosphate-Buffered Saline
PI	Propidium Iodide
PI3K	Phosphatidylinositol 3-Kinase
qRT-PCR	Quantitative Reverse Transcription Polymerase Chain Reaction
SREBP1	Sterol Regulatory Element-Binding Protein 1
STAT3	Signal Transducer and Activator of Transcription 3
