# Supplementary material for: Bufalin Suppresses Colorectal Cancer Liver Metastasis by Inhibiting De Novo Fatty Acid Synthesis via the PI3K/AKT-Mediated SREBP1/FASN Pathway
Source: Molecules. 2025 Sep 5;30(17):3634. doi: 10.3390/molecules30173634 (PMC12430088; doi:10.3390/molecules30173634)
Supplement: Supplementary file 1 [file molecules-30-03634-s001.zip › supplementary File S1-pww.pdf]

Supporting Information

Bufalin Suppresses Colorectal Cancer Liver Metastasis by Inhibiting De Novo Fatty Acid Synthesis via the PI3K/AKT-Mediated SREBP1/FASN Pathway

Wenwen Pang [1,2,†], Xiang Li [3,†], Suying Yan [4,†], Junshi Zhang [5], Ping Wu [1], Haiyang Yu [6], Bowei Zhang [3,*] and Chunze Zhang [7,8,9,*]

1 Department of Clinical Laboratory, Tianjin Union Medical Center, Nankai University, Tianjin 300071, China; wwpangscu@163.com (W.P.)
2 Tianjin Integrative Traditional Chinese and Western Medicine Oncology Institute, Tianjin 300121, China
3 School of Medicine, Nankai University, Tianjin 300071, China
4 School of Integrative Medicine, Tianjin University of Traditional Chinese Medicine, Tianjin 301617, China
5 Department of Hematology, Oncology Center, Tianjin Union Medical Center, Nankai University, Tianjin 300071, China
6 State Key Laboratory of Component-based Chinese Medicine, Tianjin University of Traditional Chinese Medicine, Tianjin 301617, China
7 Department of Colorectal Surgery, Tianjin Union Medical Center, Nankai University, Tianjin 300071, China
8 The Institute of Translational Medicine, Tianjin Union Medical Center, Nankai University, Tianjin 300071, China
9 Tianjin Institute of Coloproctology, Tianjin 300121, China
* Correspondence: bwzhang@nankai.edu.cn (B.Z.); chunze.zhang@nankai.edu.cn (C.Z.)
† These authors contributed equally to this work.

1. Primer sequence in quantitative RT-PCR

Genes	Sequence
hACTB	FORWARD：CCCTGGACTTCGAGCAAGAG
	REVERSE：CTGTGTTGGCGTACAGGTCT
hPIK3R1	FORWARD：TGGTGGCCGATGGGGAAGTG
	REVERSE：ATCTGCAAAGCGAGGGCATCTG
hAKT	FORWARD：GCCGCAGGATGTGGACCAAC
	REVERSE：ACCTTGCCGAAAGTGCCCTTG
hSREBF1	FORWARD：GTGCTCGTCTCCTTGGTGCTTC
	REVERSE：GTGCCCGCCTGTGTGCTTC
hFASN	FORWARD：CCCAATGCCTTGTTCCCACCTG
	REVERSE：GCCAGCGTCTTCCACACTATGC
mActb	FORWARD：GTGTGATGGTGGGAATGGGT
	REVERSE：ATGGCTACGTACATGGCTGG
mPik3r1	FORWARD：GCCTGGCTGGGGATCAAGAATG
	REVERSE：GGGAGTCGTTGTGCTGAACCAG
mAkt	FORWARD：GCCCACACGCTTACTGAGAACC
	REVERSE：GCACAGCCCGAAGTCCGTTATC
mSrebf1	FORWARD：TGCGGGACAGCTTAGCCTCTAC
	REVERSE：CCTCCTCCTCAGACTGCGATCC
mFasn	FORWARD：GCCGTGGTGCTGGAGATTGC
	REVERSE：AGGAGGAGCTGGAGCCGTTTG
