# Supplementary material for: Bufalin Suppresses Colorectal Cancer Liver Metastasis by Inhibiting De Novo Fatty Acid Synthesis via the PI3K/AKT-Mediated SREBP1/FASN Pathway
Source: Molecules. 2025 Sep 5;30(17):3634. doi: 10.3390/molecules30173634 (PMC12430088; doi:10.3390/molecules30173634)
Supplement: Supplementary file 1 [file molecules-30-03634-s001.zip › supplementary File S2-pww.pdf]

Supporting Information

Bufalin Suppresses Colorectal Cancer Liver Metastasis by Inhibiting De Novo Fatty Acid Synthesis via the PI3K/AKT-Mediated SREBP1/FASN Pathway

Wenwen Pang [1,2,†], Xiang Li [3,†], Suying Yan [4,†], Junshi Zhang [5], Ping Wu [1], Haiyang Yu [6], Bowei Zhang [3,*] and Chunze Zhang [7,8,9,*]

[1] Department of Clinical Laboratory, Tianjin Union Medical Center, Nankai University, Tianjin 300071, China; wwpangscu@163.com (W.P.)
[2] Tianjin Integrative Traditional Chinese and Western Medicine Oncology Institute, Tianjin 300121, China
[3] School of Medicine, Nankai University, Tianjin 300071, China
[4] School of Integrative Medicine, Tianjin University of Traditional Chinese Medicine, Tianjin 301617, China
[5] Department of Hematology, Oncology Center, Tianjin Union Medical Center, Nankai University, Tianjin 300071, China
[6] State Key Laboratory of Component-based Chinese Medicine, Tianjin University of Traditional Chinese Medicine, Tianjin 301617, China
[7] Department of Colorectal Surgery, Tianjin Union Medical Center, Nankai University, Tianjin 300071, China
[8] The Institute of Translational Medicine, Tianjin Union Medical Center, Nankai University, Tianjin 300071, China
[9] Tianjin Institute of Coloproctology, Tianjin 300121, China
* Correspondence: bwzhang@nankai.edu.cn (B.Z.); chunze.zhang@nankai.edu.cn (C.Z.)
† These authors contributed equally to this work.

1. Original data output from the flow cytometer

(a)

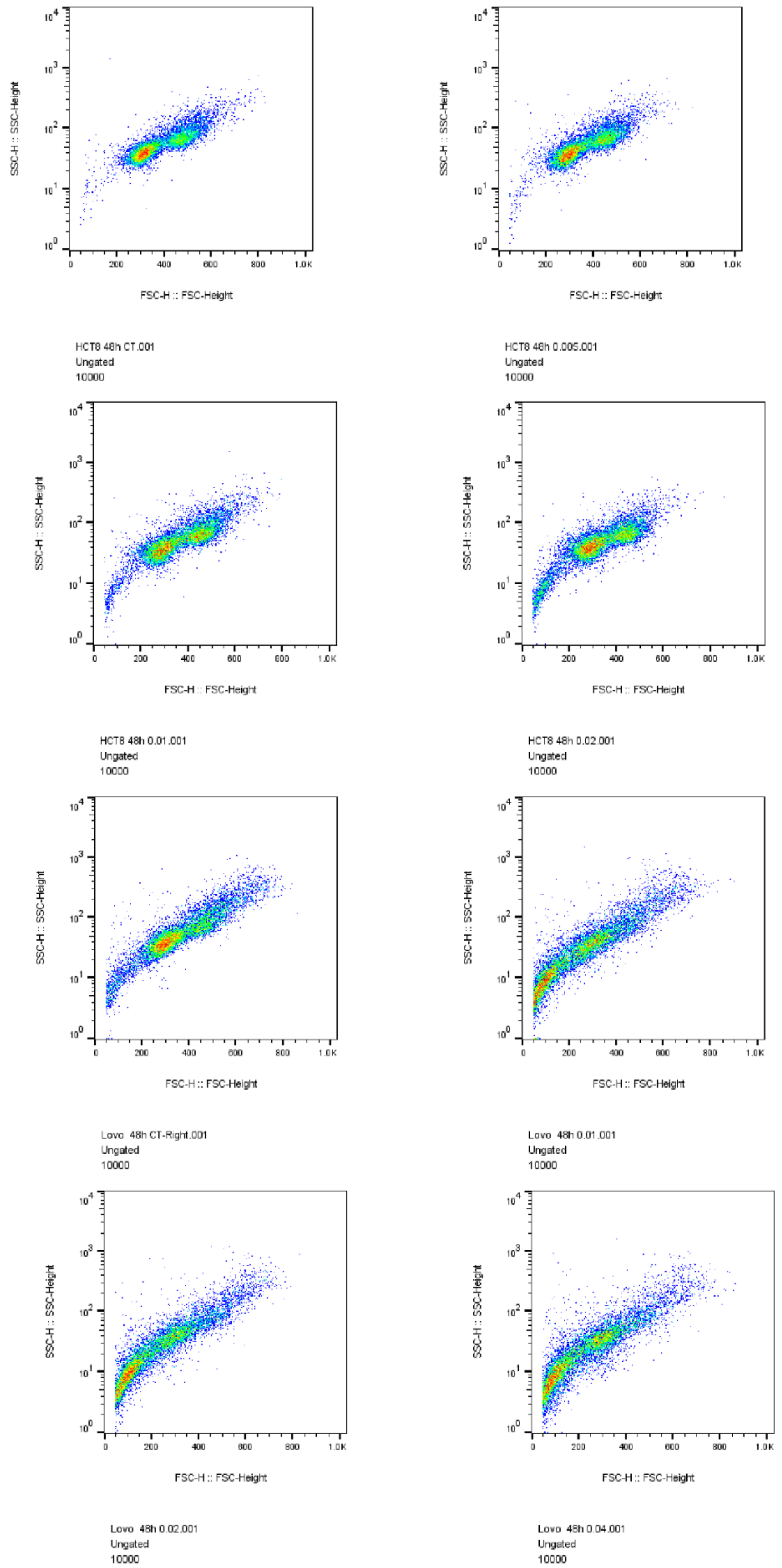

Figure S1: Flow cytometry original of the cell cycle.

(b)

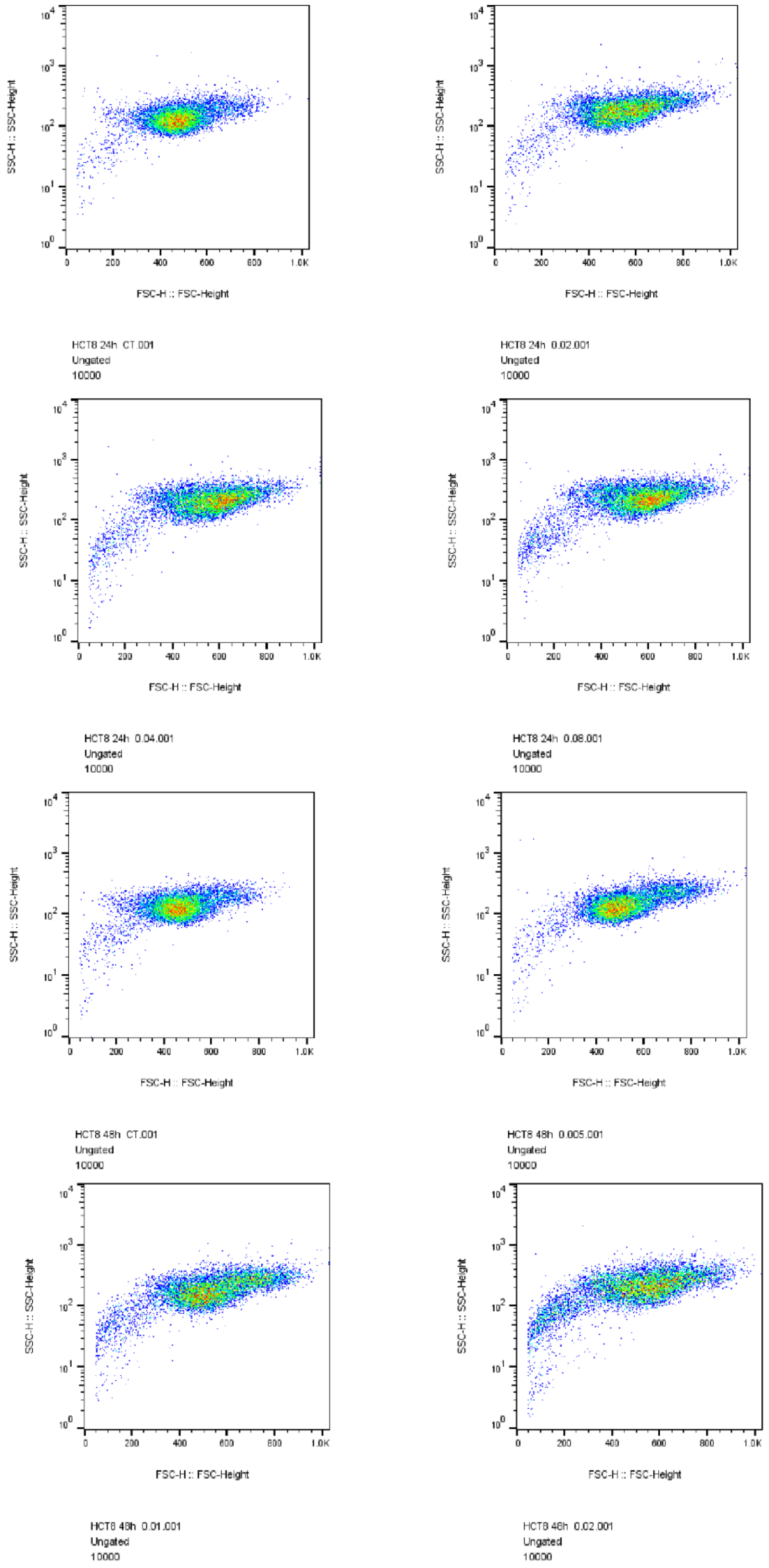
SSC-H :: SSC-Height
FSC-H :: FSC-Height
HCT8 24h CT.001
Ungated
10000
HCT8 24h 0.02.001
Ungated
10000
HCT8 24h 0.04.001
Ungated
10000
HCT8 24h 0.08.001
Ungated
10000
HCT8 48h CT.001
Ungated
10000
HCT8 48h 0.005.001
Ungated
10000
HCT8 48h 0.01.001
Ungated
10000
HCT8 48h 0.02.001
Ungated
10000

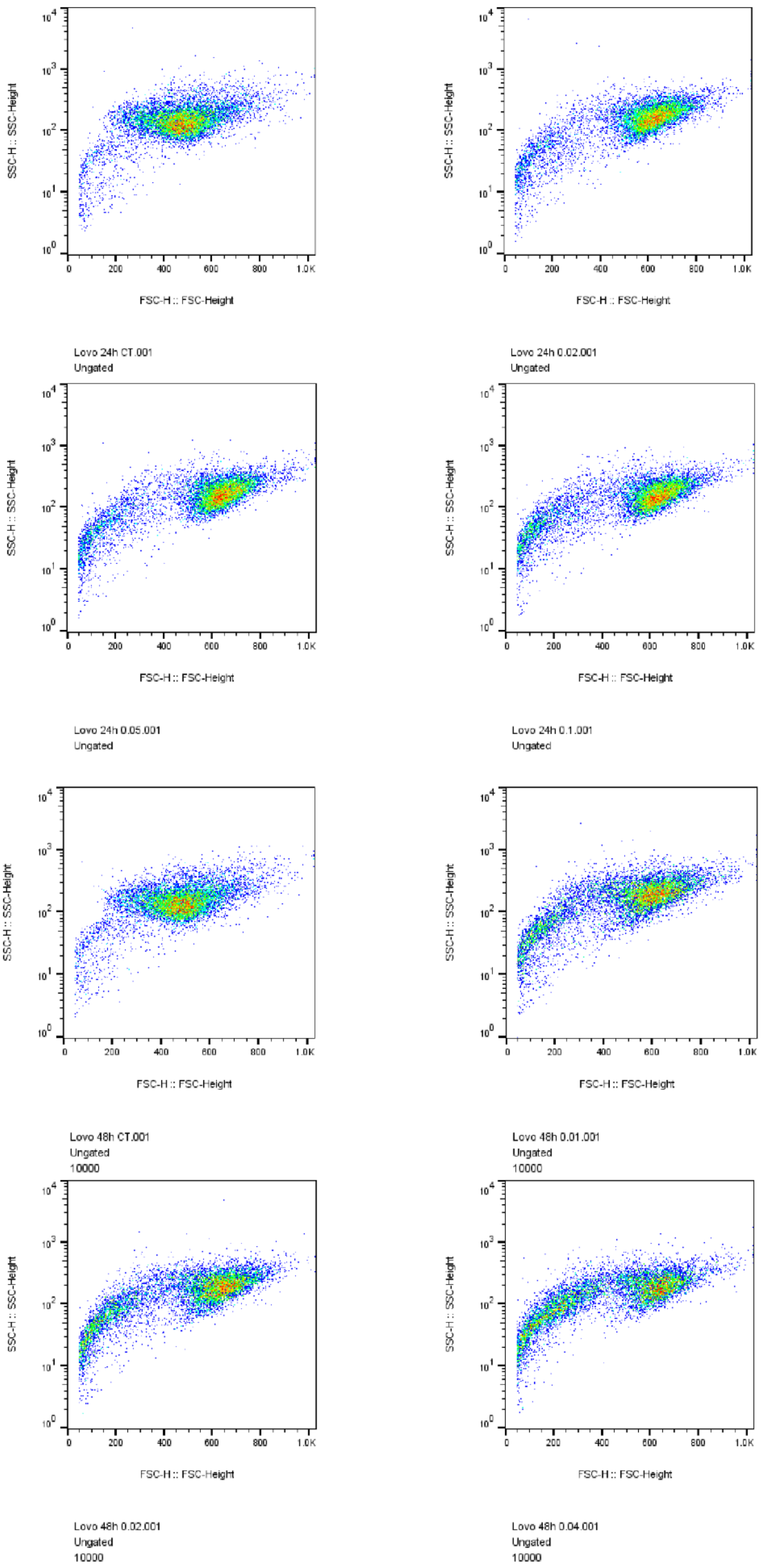

Figure S2: Flow cytometry image of apoptosis.
